# Supplementary material for: Clinical Outcome and Prognostic Factors of Pancreatic Adenosquamous Carcinoma Compared to Ductal Adenocarcinoma—Results from the German Cancer Registry Group
Source: Cancers (Basel). 2022 Aug 16;14(16):3946. doi: 10.3390/cancers14163946 (PMC9406158; doi:10.3390/cancers14163946)
Supplement: Supplementary file 1 [file cancers-14-03946-s001.zip › cancers-1873728-supplementary.pdf]

Supplementary Figure S1

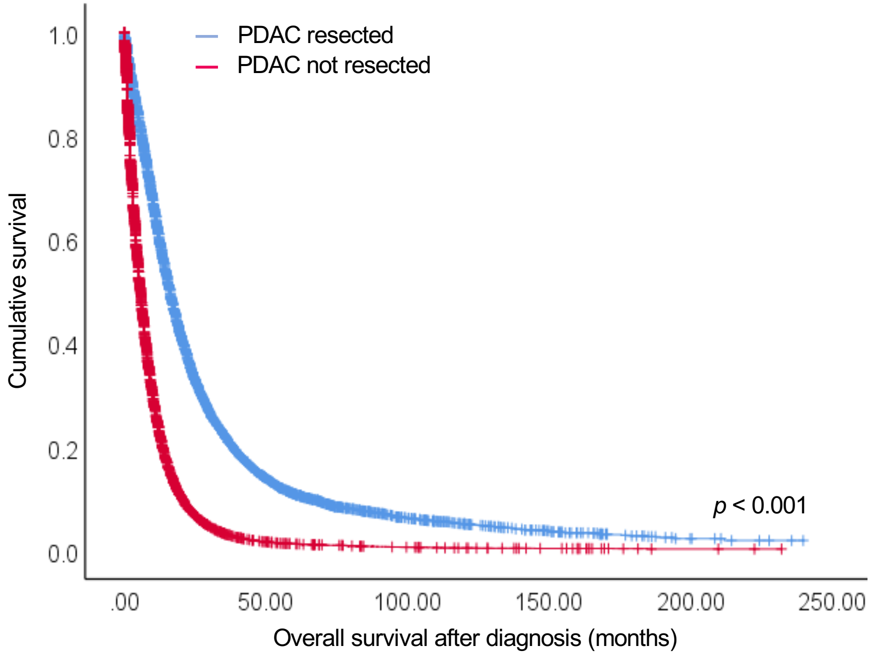

Supplementary Figure S1: Survival curve of resected PDAC patients compared to not resected PDAC patients. Median overall survival of resected PDAC patients (16.17 months; 95% CI 15.78 – 16.55 months) was significantly longer compared with unresected PDAC patients (5.73 months; 95% CI 5.62 – 5.84 months).

Supplementary Figure S2

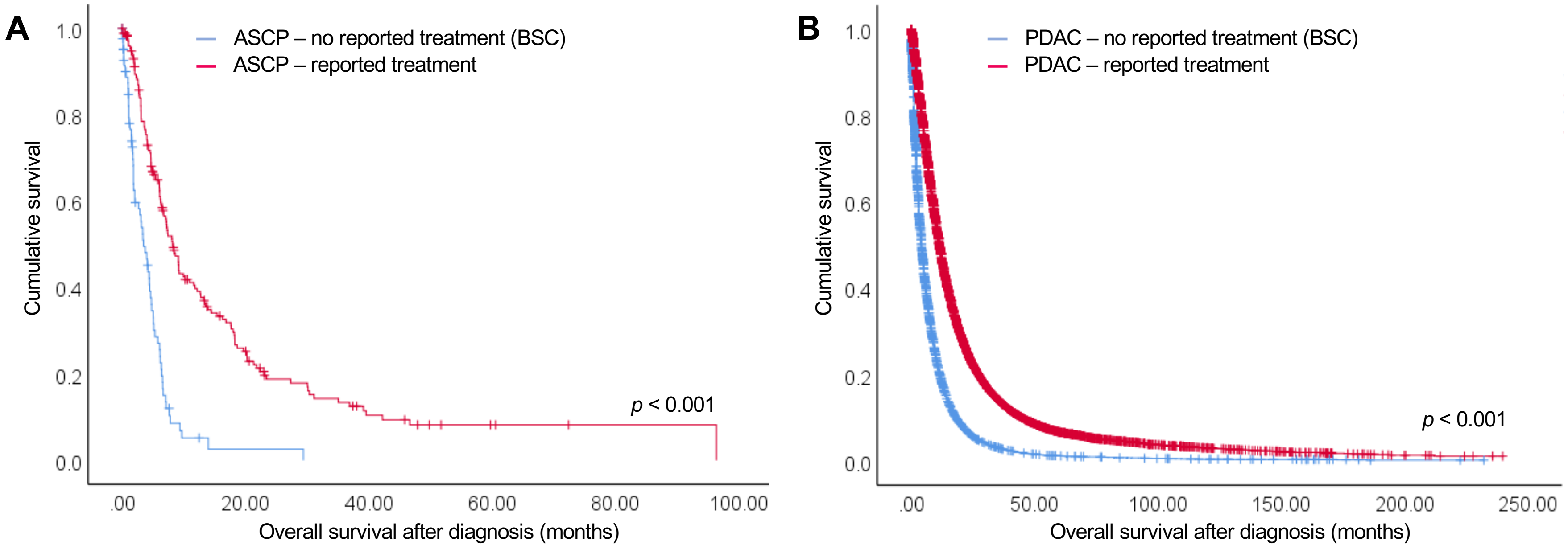

Supplementary Figure S2: Survival curves of (A) ASCP and (B) PDAC patients with and without reported treatment (best supportive care, BSC). Median overall survival in the subgroup of patients with no reported specific cancer treatment compared to those with reported treatment was significantly shorter with (A) only 3.47 (95% CI 2.35 – 4.59) compared to 8.13 (95% CI 6.74 – 9.53) for ASCP patients and (B) 4.1 (95% CI 3.97 – 4.23) compared to 11.3 (95% CI 11.10 – 11.50) months for PDAC patients.

Supplementary Table S1

Parameter	ASCP		PDAC		ASCP + PDAC	
	HR (95% CI)	p-value	HR (95% CI)	p-value	HR (95% CI)	p-value
Histology (ASCP vs PDAC)					1.303 (1.013-1.677)	0.039
Sex (male vs female)	0.867 (0.497-1.512)	0.165	1.048 (0.986-1.114)	0.954	1.044 (0.982-1.109)	0.166
Age (> 65 years vs ≤ 65)	1.399 (0.764-2.562)	0.267	1.265 (1.186-1.348)	<0.001	1.265 (1.187-1.348)	<0.001
Distant metastases (M1 vs M0)	3.436 (1.354-8.856)	0.010	1.614 (1.453-1.793)	<0.001	1.625 (1.464-1.804)	<0.001
T-stage (T4 vs T2/T3)	0.407 (0.49-3.356)	0.404	1.013 (0.848-1.209)	0.890	0.997 (0.836-1.190)	0.976
Lymph node metastases (N+ vs N0)	1.115 (0.590-2.105)	0.738	1.499 (1.392-1.615)	<0.001	1.497 (1.391-1.611)	<0.001
Lymph vessel invasion (L1 vs L0)	1.313 (0.744-2.316)	0.384	1.169 (1.089-1.254)	<0.001	1.172 (1.093-1.256)	<0.001
Blood vessel invasion (V1 vs V0)	1.793 (0.957-3.359)	0.068	1.160 (1.078-1.248)	<0.001	1.166 (1.084-1.254)	<0.001
Resection margin status (R+ vs R0)	1.711 (0.974-3.007)	0.062	1.487 (1.388-1.592)	<0.001	1.487 (1.389-1.592)	<0.001
Grading (G3/G4 vs G1/G2)	0.942 (0.509 -1.744)	0.850	1.412 (1.327-1.502)	<0.001	1.405 (1.321-1.495)	<0.001
Adjuvant therapy (no vs yes)	5.361 (2.858-10.055)	<0.001	1.871 (1.758-1.990)	<0.001	1.894 (1.781-2.014)	<0.001

Supplementary Table S1: Multivariate Cox regression analysis for survival of resected ASCP and PDAC patients including all parameters and compared groups. ASCP – adenosquamous carcinoma of the pancreas; CI – confidence interval; HR – hazard ratio; PDAC – pancreatic ductal adenocarcinoma.

Supplementary Table S2

Parameter	ASCP				PDAC				ASCP + PDAC			
	N	Deaths	Median survival in months (95% CI)	p-value	N	Deaths	Median survival in months (95% CI)	p-value	N	Deaths	Median survival in months (95% CI)	p-value
Overall	134	100	11.80 (8.20-15.40)		11965	9136	16.17 (15.78-16.55)		12099	2863	16.17 (15.79-16.55)	
Histology												
ASCP									134	100	11.80 (8.20-15.40)	0.007
PDAC									11965	9136	16.17 (15.78-16.55)	
Sex												
male	76	61	9.16 (3.21-15.13)	0.998	6374	4900	15.47 (14.99-15.95)	0.051	6450	4961	15.40 (14.92-15.88)	0.049
female	58	39	12.73 (7.48-17.98)		5591	4236	16.63 (16.07-17.20)		5649	4275	16.57 (16.00-17.13)	
Age (years)												
≤65	49	36	13.23 (3.75-22.72)	0.497	4686	3517	18.40 (17.66-19.14)	<0.001	4735	3553	18.37 (17.64-19.09)	<0.001
>65	85	64	9.17 (3.57-14.77)		7279	5619	14.43 (13.99-14.88)		7364	5683	14.40 (13.96-14.84)	
Distant metastasis												
M0	111	78	13.43 (7.22-19.65)	0.002	9499	6962	18.40 (17.89-18.91)	<0.001	9610	7040	18.37 (17.87-18.86)	<0.001
M1	19	18	6.03 (3.85-8.21)		1784	1606	7.20 (6.69-7.71)		1803	1624	7.20 (6.70-7.70)	
T-stage												
pT0	0	0			23	7	56.53 (0.00-126.69)	<0.001	23	7	56.53 (0.00-126.69)	<0.001
pT1	0	0			406	259	34.17 (28.38-39.95)		406	259	34.17 (28.38-39.95)	
pT2/3	127	95	12.17 (8.60-15.73)	0.979	10303	7877	16.33 (15.92-16.75)		10430	7972	16.30 (15.89-16.72)	
pT4	6	4	6.07 (1.23-10.91)		829	737	8.90 (8.15-9.65)		835	741	8.83 (8.11-9.56)	
Lymph node metastases												
N0	40	28	14.43 (8.38-20.49)	0.064	3610	2491	22.20 (21.18-23.22)	<0.001	3650	2519	22.10 (21.08-23.12)	<0.001
N+	93	71	9.17 (5.01-13.32)		8020	6371	14.23 (13.82 - 14.64)		8113	6442	14.23 (13.82-14.64)	
Lymph vessel invasion												
L0	45	29	18.67 (15.85-21.48)	0.096	3252	1975	22.83 (21.79-23.88)	<0.001	3297	2004	22.63 (21.59-23.68)	<0.001
L1	66	48	9.23 (4.26-14.20)		4850	3642	15.87 (15.30-16.43)		4916	3690	15.80 (15.24-16.36)	
Blood vessel invasion												
V0	70	49	17.63 (13.43-21.84)	0.031	5871	3947	19.93 (19.29-20.58)	<0.001	5941	3996	19.83 (19.19-20.48)	<0.001
V1	40	28	8.10 (4.89-11.306)		1958	1440	13.47 (12.70-14.24)		1998	1468	13.27 (12.50-14.03)	
Resection margin status												
R0	75	53	13.50 (9.97-17.03)	0.369	6089	4162	21.00 (20.31-21.69)	<0.001	6164	4215	20.87 (20.18-21.55)	<0.001
R+	28	19	9.17 (0.00-28.13)		2355	1793	13.23 (12.61-13.85)		2383	1812	13.23 (12.60-13.86)	
Grading												
G1/G2	38	27	11.80 (2.26-21.34)	0.508	6021	4397	19.87 (19.21-20.52)	<0.001	6059	4424	19.83 (19.18-20.48)	<0.001
G3/G4	89	66	10.90 (4.50-17.30)		4943	3956	12.60 (12.16-13.04)		5032	4022	12.60 (12.16-13.04)	
Adjuvant Therapy												
Yes	48	30	22.37 (14.48-30.25)	<0.001	4973	3525	21.57 (20.92-22.22)	<0.001	5021	3555	21.57 (20.92-22.21)	<0.001
No	86	70	6.03 (4.56-7.51)		6992	5611	12.17 (11.71-12.62)		7078	5681	12.07 (11.62-16.55)	

Supplementary Table S2: Survival analysis including all parameters and compared groups of ASCP and PDAC patients. ASCP – adenosquamous carcinoma of the pancreas; CI – confidence interval; N – number; PDAC – pancreatic ductal adenocarcinoma.
